# Application of decellularized vascular matrix in small-diameter vascular grafts

**DOI:** 10.3389/fbioe.2022.1081233

**Published:** 2023-01-06

**Authors:** Yuanming Li, Ying Zhou, Weihua Qiao, Jiawei Shi, Xuefeng Qiu, Nianguo Dong

**Affiliations:** Department of Cardiovascular Surgery, Union Hospital, Tongji Medical College, Huazhong University of Science and Technology, Wuhan, China

**Keywords:** small-diameter vascular grafts, decellularized vascular matrix, tissue-engineered vascular grafts, design criteria, decellularization technologies, commercialization

## Abstract

Coronary artery bypass grafting (CABG) remains the most common procedure used in cardiovascular surgery for the treatment of severe coronary atherosclerotic heart disease. In coronary artery bypass grafting, small-diameter vascular grafts can potentially replace the vessels of the patient. The complete retention of the extracellular matrix, superior biocompatibility, and non-immunogenicity of the decellularized vascular matrix are unique advantages of small-diameter tissue-engineered vascular grafts. However, after vascular implantation, the decellularized vascular matrix is also subject to thrombosis and neoplastic endothelial hyperplasia, the two major problems that hinder its clinical application. The keys to improving the long-term patency of the decellularized matrix as vascular grafts include facilitating early endothelialization and avoiding intravascular thrombosis. This review article sequentially introduces six aspects of the decellularized vascular matrix as follows: design criteria of vascular grafts, components of the decellularized vascular matrix, the changing sources of the decellularized vascular matrix, the advantages and shortcomings of decellularization technologies, modification methods and the commercialization progress as well as the application prospects in small-diameter vascular grafts.

## 1 Introduction

The incidence of cardiovascular disease (CVD) has been increasing worldwide since the beginning of the 21st century, becoming one of the prominent causes of mortality, and has affected approximately 523 million people (Roth et al., 2020). In the European Union, more than 3.9 million people have died because of CVD, accounting for 45% of the total annual death toll (European Heart Network, 2017). Moreover, each year 11.3 million new cases of CVD are reported in the European Union (Movsisyan et al., 2020; World Health Organization, 2020). Although percutaneous coronary intervention and other traditional operations are widely used, patients with severe coronary atherosclerotic heart disease need to undergo vascular transplantation, such as coronary artery bypass grafting (CABG). When the arteriovenous vascular lesions have progressed to an end-stage, conservative therapy or conventional surgery cannot effectively cure patients. In the United States alone, approximately 4,50,000 patients undergo vascular bypass surgery each year (Kim et al., 2015; Benjamin et al., 2017), concomitantly increasing the demand for vascular grafts and broadening its application prospects.

Vascular grafts are generally of three types: allogeneic, autologous, and synthetic. Allogeneic vascular grafts often lead to immune rejection, while autologous vascular grafts, such as the internal thoracic artery and saphenous vein, are the best choices for patients undergoing CABG. Nevertheless, on account of peripheral vascular diseases and other reasons, not all patients have good autologous blood vessels to meet the requirements of autologous transplantation. Among synthetic vascular grafts, various polymer materials such as polylactic acid, polyglycolic acid (PGA), and expanded polytetrafluoroethylene (ePTFE) have become alternatives to autologous vessels. Nonetheless, they have many deficiencies in biocompatibility and vascular compliance. For instance, polymer materials are cytotoxic to a certain extent and can easily cause a rejection reaction. Their degradation rate also limits the clinical application. Therefore, the field of tissue engineering has emerged, capable of producing different vascular grafts.

In recent years, tissue engineering has become a topic of interest in biomedical research, especially in the cardiovascular field. It utilizes advanced technologies for the preparation of vascular grafts with good biological activity and biocompatibility. Tissue-engineered vascular grafts (TEVGs) are broadly of three categories: large-diameter vascular grafts with a diameter >8 mm, medium-diameter vascular grafts with a 6–8 mm diameter, and small-diameter vascular grafts with a diameter <6 mm. Among them, the initial application of small-diameter vascular grafts involves CABG. Large and medium-diameter TEVGs exhibit high blood flow and low vascular resistance; therefore, the patency rate is ideal and can meet clinical needs. A variety of high-performance synthetic materials have been successfully used in the production of TEVGs with large or medium diameters that have performed well. On the contrary, when the blood in small-diameter vascular grafts flows through the inner wall, the likelihood of platelet attachment and thrombus formation increases. Meanwhile, the vascular graft undergoes remodeling post-implantation. The mismatch of mechanical properties between the graft and the natural blood vessel also leads to the excessive proliferation of smooth muscle cells like stromal cells, forming new intima hyperplasia (Mallis et al., 2020a).

Among small-diameter vascular grafts, the decellularized vascular matrix is one of the most popular materials attributed to its superior biocompatibility and non-immunogenicity. The extracellular matrix is the component of the decellularized vascular matrix, which refers to the removal of immunogenic cell components through decellularization technologies of blood vessels from animals or humans and TEVGs. The ultrastructure of the extracellular matrix is well preserved while the cellular structure is exenterated; as a consequence, its structure is more similar to that of the natural vessel wall. In view of the above advantages, the decellularized vascular matrix is expected to be a more promising and efficiently performing vascular scaffold material with good performance that satisfies clinical demands.

This review elaborately introduces the six aspects of the decellularized vascular matrix. First, we introduce the design criteria for vascular grafts. Second, the components of the decellularized vascular matrix are analyzed. Third, with the development in recent years, the source of the decellularized vascular matrix has changed. Thus, the advantages and shortcomings of different decellularization technologies have been compared, and the evaluation criteria of decellularization are described. In addition, the different modification methods used to improve the performance and long-term patency rate are listed. Lastly, we summarize the commercialization process of the decellularized vascular matrix and its potential application in small-diameter vascular grafts.

## 2 Criteria for vascular graft design

A set of standardized design criteria have been developed after years of attempts to manufacture vascular grafts. These criteria are outlined in ISO 7198:2017, “Cardiovascular implants and extracorporeal systems—tubular vascular grafts and vascular patches” (Moore et al., 2022). The required mechanical, biological, and immunological properties of suitable vascular grafts as well as relevant definitions are discussed below (Table 1).

**TABLE 1:** Relevant parameters for the properties of vascular grafts.

Parameter	Definition	Test methods
Burst pressure	The pressure at which one end of the vascular graft is closed and liquid is slowly and uniformly injected from the other end until the vessel expands and ruptures	Bursting tester
Suture retention strength	Defined as the peak strength when the surgical wire is pulled out the wall of the tube	Universal tensile tester
Ultimate tensile strength	The maximum stress that the vascular graft can withstand when stretched before rupture	Universal tensile tester
Compliance	The ability of the vascular graft to expand radially due to internal pressure	Compliance tester
Patency	After vascular implantation, the grafts remained unobstructed without any intervention	Ultrasound

### 2.1 Mechanical properties of vascular grafts

The mechanical properties of vascular grafts should be as close as possible to those of the internal mammary artery, the obvious choice for a natural artery. In patients with severe hypertension, the systolic pressure may be as high as 180 mmHg, while the burst pressure of natural blood vessels exceeds 3,000 mmHg. According to the bioengineering consensus, the graft with burst pressure (>1,000 mmHg) is desirable (L'Heureux et al., 2006; Konig et al., 2009). Concurrently, compliance should match as much as possible to those of the natural blood vessels. The suture retention strength exceeds 1 N, and the ultimate tensile strength is higher than 1 MPa, with the compliance being 10%–20%/100 mmHg (Girerd et al., 1992; L'Heureux et al., 2006; Konig et al., 2009; Stekelenburg et al., 2009; Seifu et al., 2013). A sufficient extracellular matrix can provide appropriate ultimate tensile strength, suture retention strength, and fracture strength for vascular grafts.

### 2.2 Biological properties of vascular grafts

To ensure vascular patency, the lumen surface of vascular grafts should have proper blood compatibility to prevent or reduce the adhesion of platelets and the activation of the coagulation system; this is a necessary condition, especially for small-diameter vascular grafts. Likewise, vascular grafts also need excellent cell compatibility on the lumen surface for cell adhesion and proliferation. If there are endothelial cells—from autologous veins or circulating progenitor cells—on the lumen surface, their presence reduces the probability of thrombosis. Tissue engineering can promote early endothelialization of the vascular wall to thus improve the biocompatibility of vascular grafts. Moreover, vascular grafts need to be able to be remodeled as part of the body after implantation. Only when smooth muscle cells and endothelial cells proliferate, the structure and function of vascular grafts can gradually approach the natural blood vessels.

### 2.3 Immunological properties of vascular grafts

The extracellular matrix of vascular grafts needs to be human-derived to minimize the risk of inflammation, foreign body reaction, and immune recognition. If the cells are not autogenous and have not been completely removed, the recipient’s immune system recognizes antigens from the donor’s graft surface after implantation, triggering an autoimmune response. Vascular grafts can be divided into allografts and xenografts depending on the cell source.

The antigens that cause allograft rejection are histocompatibility antigens. Among them, the histocompatibility antigen that can cause strong rejection is called major histocompatibility antigen. The histocompatibility antigen that causes weak rejection is called secondary histocompatibility antigen (mH antigen). The key to successful transplantation depends on whether the tissue capacitive antigens between the donor and recipient are consistent or similar. After transplantation, T cells of the recipient recognize MHC antigen on the surface of the graft through direct and indirect ways to cause the immune response. Human leukocyte antigen (HLA), encoded by the major histocompatibility complex (MHC), is the most important antigen of allograft rejection (Hamada et al., 2021). Especially the antigen molecules located in the HLA-DR, followed by HLA-A, HLA-B, HLA-DQ, and HLA-DP (Hubscher et al., 1990; Fleischhauer et al., 2001; Lim et al., 2016; Wiebe et al., 2017; Féray et al., 2021). There was no significant relationship between HLA-C and transplant rejection (Piccinni et al., 2021).

The biggest problem caused by xenotransplantation is immune rejection, especially hyperacute rejection. Hyperacute rejection can kill cells from the donor vascular graft within 24 h (Tector et al., 2020). The reason is that natural antibodies in human blood bind to antigens on the surface of the xenograft to activate the complement system. When activated, the complement system forms membrane-attacking complexes, triggering a series of chain reactions including endothelial cell dysfunction, platelet aggregation and thrombosis, leading to transplantation failure. Xenograft immunogenicity is directly related to the presence of several antigens including the α-Gal epitope, the linked N-glycolyl neuraminic sialic acid (Neu5Gc) and the Sd(a) (Byrne et al., 2011; Naso et al., 2013; Gao et al., 2017). Mammals have these antigenic molecules except for humans, apes and certain monkeys.

Consequently, the decellularized vascular matrix can only be used as a vascular graft in the clinical application if it meets all the above-discussed criteria. In general, these factors should also consider market regulation, large-scale production, and other requirements. So far, this goal has not been achieved and is still in progress.

## 3 Components of decellularized vascular matrix

The decellularized vascular matrix refers to the non-cellular part of blood vessel wall. It often includes an intricate 3D structure and is filled with an invisible gelatinous structure. In the past, it was considered as a simple supporting structure of blood vessels. Recent discoveries have completely overturned this old view. The decellularized vascular matrix is not only a supporting structure, but also a microenvironment for cell life. It is closely related to cell survival, regeneration, repair, and immunity (Xing et al., 2020; Kotla et al., 2021; Kretschmer et al., 2021).

**FIGURE 1:**
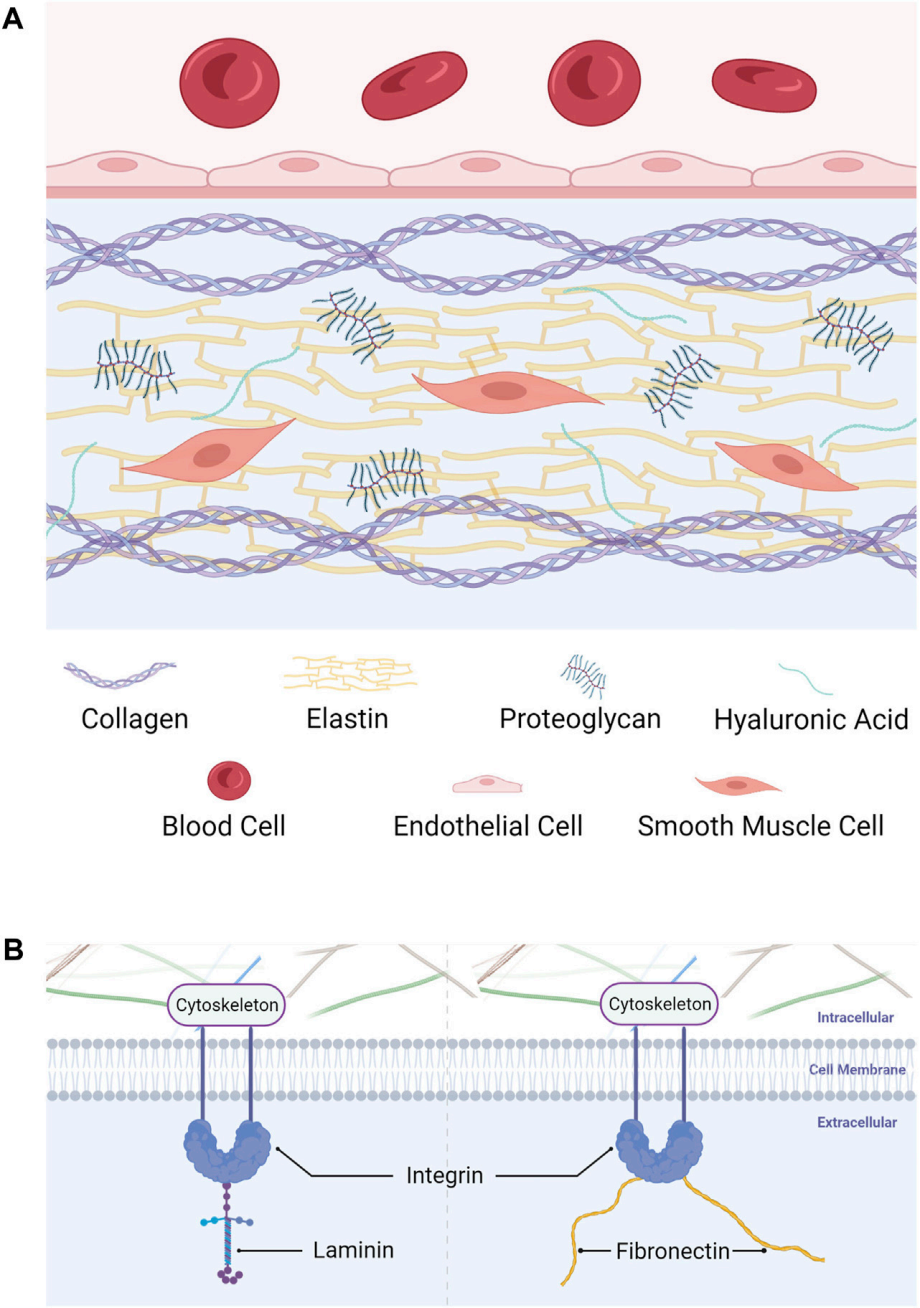
Components of the decellularized vascular matrix. **(A)** Structural protein and invisible gelatinous structures. **(B)** Linked/adhesive protein.

The composition of decellularized vascular matrix varies in different functional and developmental stages of blood vessels. For example, the content of elastin in the vascular matrix of arteries is much higher than that of veins. Besides, fibronectin and collagen IV decreased with age, while collagen I, collagen III and laminin increased with age (Williams et al., 2014). Considering the source and application of vascular grafts, this article only focuses on the main components of mammalian natural blood vessels. Simply stated, the decellularized vascular matrix consists of three components: structural protein, linked/adhesive protein, and invisible gelatinous structures. Specifically, structural proteins mainly include collagen and elastin. The linked/adhesive proteins mainly include integrin, fibronectin and laminin. Invisible gelatinous structures mainly include proteoglycan and hyaluronic acid (Figure 1).

### 3.1 Structural proteins

#### 3.1.1 Structural protein refers to the fibrous protein in the decellularized vascular matrix, which forms a 3D structure

Collagen is the most important protein of the decellularized vascular matrix. This component is also the most closely related to vascular remodeling. Collagen constitutes fibers that form the three-dimensional structure of the extracellular matrix, provide living space for cells, and support the physiological morphology of vessels (Pankov and Yamada, 2002). On the other hand, the three-dimensional structure constructed using collagen can regulate cell adhesion, proliferation, and differentiation, as well as the immune response of immune cells through biological force transduction, thus regulating vascular remodeling and regeneration (Chu et al., 2017; Velez et al., 2019). Years of research have led to the discovery of 28 kinds of collagens (Bielajew et al., 2020). According to their molecular structure and function, collagens are further classified into fiber collagen, fiber bound collagen, basement membrane collagen, long chain collagen, filamentous collagen, short chain collagen, multi-cluster collagen, and transmembrane collagen. These several types of collagens can perform their roles, resemble the actual microenvironment of natural blood vessels and play a better role in repair and regeneration.

Elastin is the main component of elastic fibers in the extracellular matrix. The fibers with elastin can provide elastic recoil for blood vessels (Vindin et al., 2019). The high content of hydrophobic amino acids in elastin makes it one of the proteins with the strongest chemical resistance, rendering it with reversible ductility in the cyclic loading process (Rosenbloom et al., 1993). Elastin plays a key role in cell adhesion and migration and participates in intracellular signaling pathways in blood as well (Oleggini et al., 2007). In the vessel development process, the assembly of elastin into elastin fibers changes with the vessels’ maturation and aging (Robert et al., 1984). In aging vessels, matrix metalloproteinases participate in elastin decomposition, resulting in a significant decrease in its content and, consequently, the elasticity of blood vessel walls (Hynes and Naba, 2012).

### 3.2 Linked/adhesive proteins

Linked/adhesive proteins refers to a series of proteins with adhesiveness and connectivity. They are usually spherical, responsible for connecting the fibrous protein structure in the decellularized vascular matrix with cells.

Integrin is one of the most important globular proteins that serve multiple functions. For example, as a “bond,” integrin connects the cytoskeleton to the vascular matrix. This connection not only stabilizes cells, but also allows vascular cells to feel the changes of external mechanical information (such as pressure), thus regulating the biological function of cells (Sun et al., 2016). In addition, integrin, as a receptor, can realize the information transmission between vascular matrix and cells (Singh et al., 2010).

Fibronectin is widely distributed and has the ability to combine with a variety of vascular matrix components. The multiple structures of fibronectin enable it to simultaneously combine with cell surface receptors (such as integrin), collagen, proteoglycans, and other adhesion molecules (Nelson and Bissell, 2006; Barkan et al., 2010). This property also enables it to mediate the assembly of a variety of extracellular matrix proteins. In short, fibronectin thus shapes the matrix structure and plays a connecting role (Avila Rodriguez et al., 2018).

Laminin is the main component of basement membrane. The *α*, *β*, and *γ* peptide chains are interlinked to form a cross structure. It is closely connected with basal cells to fix them. Laminin affects adhesion, differentiation, migration and phenotype stability, and provides resistance to apoptosis of related cells (Domogatskaya et al., 2012).

### 3.3 Invisible gelatinous structures

In addition to the components mentioned above, there are proteoglycans, glycoproteins, amino polysaccharides, proteases, bioactive molecules, electrolytes, and water in invisible gelatinous structures of the decellularized vascular matrix.

Proteoglycan is a huge molecule with a protein chain as the core and glycosaminoglycans (GAGs) as the side branches. Because GAGs can attract water, it forms a gelatinous structure to fill the vascular matrix (Scott and Panitch, 2013). This gel maintains the stability of microenvironment, provides nutrition for cells and transmit signals (Koyama et al., 1998).

Hyaluronic acid is a huge single chain molecule only composed of disaccharides. It can combine with many proteoglycans to form a huge proteoglycan polymer and work together with elastin and collagen to resist damage to tissues and body caused by external impact (Li et al., 2020; Li et al., 2021).

None of the components are isolated. Collagen and elastin provide mechanical strength and flexibility to the decellularized vascular matrix. Linked/adhesive proteins participate in mechanical information transmission, act as receptors to transmit chemical information and regulate the activity of biological molecules. Invisible gelatinous structures can transmit signals, store important bioactive substances, and endow blood vessels with flexibility and buffering capacity against external forces. All of these components play indispensable roles in creating the most suitable environment for the survival of cells and the body. Thus, the decellularized vascular matrix retains the complex extracellular three-dimensional structure and biological activity, much like the structure and function of the native vessels. Undoubtedly, it is more in line with the standards of TEVGs and has broad application prospects.

## 4 Sources of decellularized vascular matrix

**FIGURE 2:**
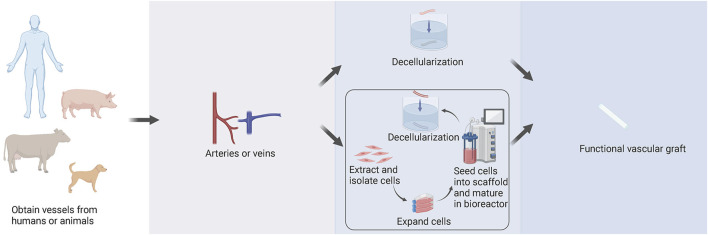
Sources of the decellularized vascular matrix. There are two main methods to create the decellularized vascular matrix by obtaining vessels from humans or animals. Vessels are either directly processed by decellularization technologies, or cells can be extracted and isolated from vessels, including smooth muscle cells, fibroblasts, and endothelial cells. With culture and amplification *in vitro*, cells are seeded into a degradable polymer scaffold and then matured in a bioreactor. After a period, the scaffold degrades, and the decellularized vascular matrix is obtained by decellularization technologies.

There are two main sources of the decellularized vascular matrix, including directly obtained from animals or humans and artificially prepared by tissue engineering (Figure 2). The sources of the decellularized vascular matrix are constantly evolving and are being updated through experimental techniques.

### 4.1 Vascular matrices prepared by decellularization of obtained vessels

Early protocols regarding the preparation of decellularized vascular matrices were mainly based on the decellularization of blood vessels obtained directly from animals or humans to obtain decellularized vascular matrices.

#### 4.1.1 Animal-derived decellularized vascular matrix

Rosenberg et al. (1966) first attempted the decellularization technique in 1966. They decellularized the bovine carotid artery and performed bypass grafting of the femoral-popliteal and iliofemoral arteries in 16 patients. However, this trial resulted in occlusion of the vascular graft postoperatively by the 2 year.

In 1995, (Sandusky et al., 1995), implanted the porcine small intestine submucosa (SIS) as a carotid artery interposition graft in dogs. No aneurism formation was found and the patency rate was equal in both the vascular graft and autogenous saphenous vein.

Katzman et al. (2005) in 2005 similarly used the mesenteric vein bioprosthesis (MVB) as a vascular graft for hemodialysis, and the patency rate of MVB was superior to that of ePTFE synthetic vascular grafts. At 12 months, the primary patency was 35.6% of MVB and 28.4% of synthetic grafts. And at 24 months, the secondary patency was 60.3% of MVB and 42.9% of synthetic.

In the same year, (Cho et al., 2005), performed carotid artery replacement in dogs. They decellularized the canine carotid arteries with .5% v/v Triton X-100, .05% v/v ammonium hydroxide and seeded the grafts with dog bone marrow mesenchymal stem cells (MSCs). Then they implanted grafts as carotid arteries interposition grafts, and their experiment demonstrated successful vascular graft remodeling *in vivo*.

Chemla and Morsy (2009) in 2009 decellularized the bovine ureter and applied it into 60 patients as arteriovenous conduits. The comparison between ePTFE and decellularized bovine ureter showed that there were no significant advantages of the decellularized bovine ureter compared with ePTFE.

In 2014, (Mancuso et al., 2014), decellularized the ovine carotid artery with 1% w/v SDS, .05% v/v Trypsin, .02% EDTA. After that, they seeded the grafts with MSCs and recellularized the grafts successfully. The histological analysis with H&E, Masson’s Trichrome, and Verhoeff van Gieson showed the ECM structure was preserved well.

Additionally, (Daugs et al., 2017), in 2017 applied the decellularization technique successfully into bovine carotid arteries with 1% w/v SD, 1% w/v CHAPS, 1% v/v Triton X-100 or .1% SDS. The histological analysis also revealed the excellent preservation of ECM structure and the results of mechanical tests showed biomechanical properties of decellularized bovine carotid arteries.

The first decellularized vascular grafts derived from bovine blood vessels and ureters. With the development of tissue engineering technology, Artecraft^®^, Solcograft^®^, ProCol^®^ (LeMaitre Vascular, Inc., Burligton, MA, United States) have been subsequently produced as vascular grafts based on these materials and put on the market today (Pashneh-Tala et al., 2016). SIS has also been proposed for the production of large and small TEVGs (Zhao et al., 2020).

#### 4.1.2 Human-derived decellularized vascular matrix

The first human-derived vascular grafts used in vascular transplantation were obtained from femoral veins from cadavers. Decellularized vessels can also be obtained from human-derived femoral arteries and iliac veins (Teebken et al., 2009; Porzionato et al., 2018). In 2005, (Madden et al., 2005), compared decellularized femoral veins from cadavers with ePTFE in a large-scale clinical trial and observed that the time required for aneurysm formation was five times longer in homogeneous vascular grafts than when using ePTFE. This demonstrated the ability of homogeneous decellularized vessels, compared to ePTFE, to reduce the likelihood of hemangioma. In 2012, (Olausson et al., 2012), decellularized iliac veins and cultured the patient’s own endothelial and smooth muscle cells, and then implanted them in patients with extrahepatic venous obstruction. These grafts were originally used as arteriovenous fistula (AVF) allografts (Wilshaw et al., 2012). Now they have been commercialized and called Synergraft^®^ (CryoLife, Inc., Kennesaw, GA, United States).

From 1974, the human umbilical vein (hUV) is being used as material for vascular bypass grafts and was subsequently commercialized as Biograft^®^ (Oblath et al., 1978; Andersen et al., 1985). In 1994, (Jarrett and Mahood, 1994), performed a large-scale clinical trial in which the human umbilical vein was used for femoropopliteal artery bypass grafts in 171 patients. The trial outcomes were a mortality rate of 6% in patients within 1-year post-surgery and a patency rate of 50% within 5 years. The results of these studies indicated that hUV may be used as a source of SDVGs production. Although the results were promising, due to major defects, hUV was stopped be applied as a vascular graft (Aalders and van Vroonhoven, 1992). HUV is technically more difficult to apply than the saphenous vein or synthetic vascular graft. In addition, hUV may be lack of elasticity and more fragile.

To attack these problems, later in 2008, (Kerdjoudj et al., 2008), performed rabbit carotid artery grafting by de-endothelializing human umbilical artery (hUA) with trypsin, and the graft was patent for more than 12 weeks. Soon in 2009, (Gui et al., 2009), performed *in vitro* hUA decellularization, leaving intact structures of the extracellular matrix, followed by *in vivo* abdominal aortic grafting. Their postoperative results suggested thrombosis of the vascular graft, although there were no vessel ruptures. In 2020, (Mallis et al., 2020b), performed similar *in vivo* and *in vitro* trials by decellularizing hUA and performing common carotid artery grafting after 30 days. These trials suggested that the vascular grafts underwent remodeling despite thrombosis and sustained the blood flow. Compared with hUV, hUA may be a better source of SDVGs. However, extended validation tests should be conducted to better determine the stability and functionality of these grafts.

### 4.2 Preparation of decellularized vascular matrix by tissue engineering

Besides the decellularization of obtained vessels, a more popular attempt is to prepare decellularized vascular matrix by tissue engineering.

In the 1970s, Charles Sparks made one of the early attempts to prepare decellularized vessels through tissue engineering to obtain the “Sparks Mandril” structure of artificial blood vessels (Sparks, 1969). The process involved subcutaneous implantation of Mandril of 5.1 mm diameter in the legs of patients, followed by covering with loosely woven synthetic polymer Dacron. A tube composed of fibrous tissue was formed outside Mandril. However, this method was abandoned because of the formation of a thrombus or aneurysm post-implantation. Weinberg and Bell published a report in 1986 announcing the *in vitro* production of the first tissue-engineered vascular graft (TEVG) (Weinberg and Bell, 1986). They cast collagen gel around the mandril supported using a polyester mesh sleeve and implanted fibroblasts into the outer layer. The tube wall was filled with smooth muscle cells, and the inner wall comprised endothelial cells. The artificial blood vessel could not be successfully implanted *in vivo* because of the low rupture strength and the inability to conduct *in vivo* tests without a polyester stent; however, this approach represents important conceptual progress. It encourages the co-culturing of other cells and endothelial cells to prepare TEVGs further.

**FIGURE 3:**
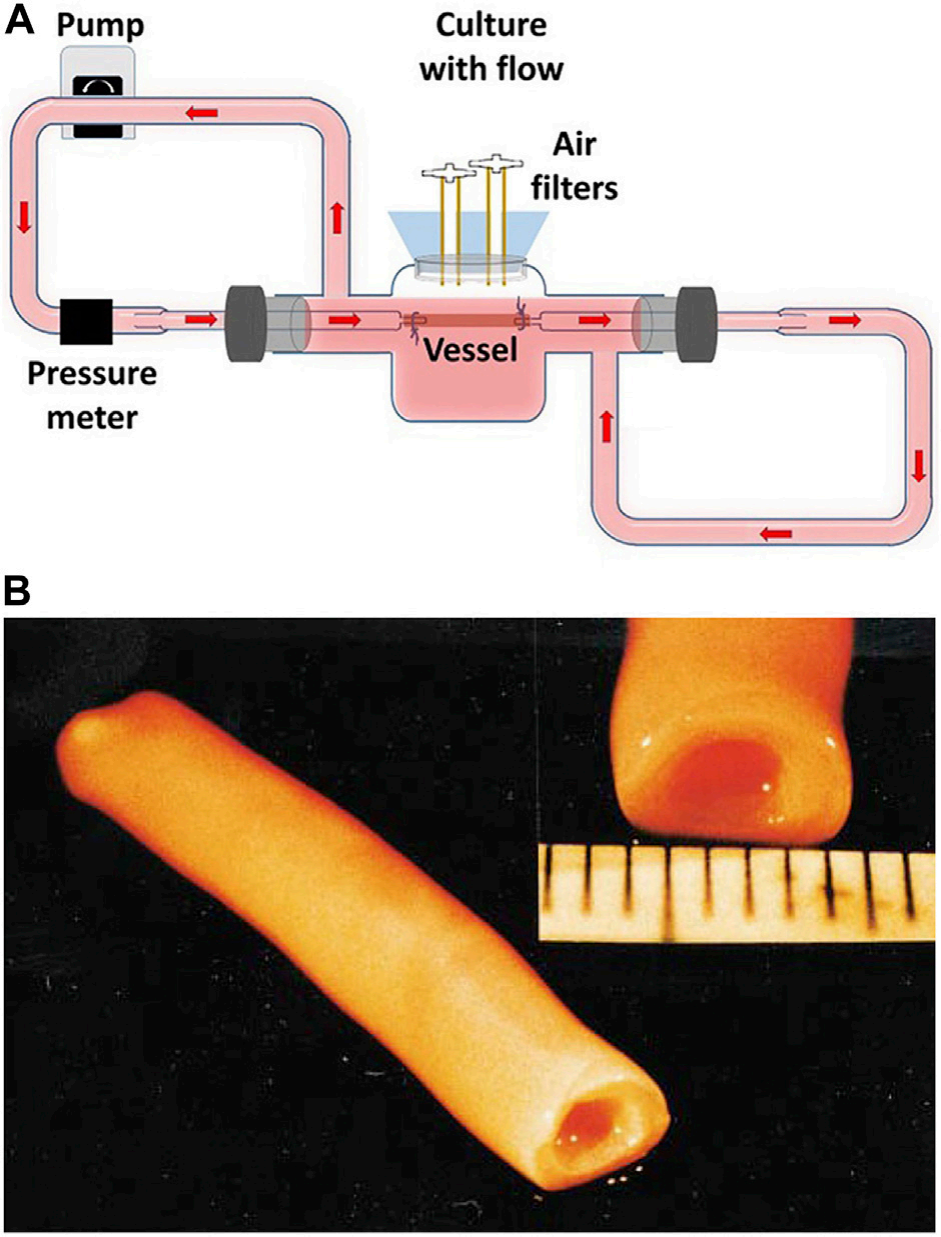
Tissue-engineered vascular graft (TEVG) in the bioreactor. **(A)** Dynamic culture of TEVG in the bioreactor (Kural et al., 2018). **(B)** Macroscopic view of a mature TEVG (L'Heureux et al., 1998).

The first case of a completely biological TEVG was proposed by L'Heureux et al. (1998). It comprised concentric layers of cell sheets, which rolled to form a tubular structure and matured in the bioreactor finally (Figure 3). First, fibroblasts and smooth muscle cells were co-cultured into monolayer cells for 30 days. The fused fibroblasts were separated from the matrix, rolled around the Mandril to form tubular structures, and then dehydrated as the intima of blood vessels. Multiple layers of smooth muscle cells were wrapped on the intima as the media layer, and the lumen was inserted into a silicone tube and placed into a bioreactor for periodic expansion. To be exact, the bioreactor was filled with culture medium and equipped with a pulsatile pump instead of heart. Pulsatile radial stress was applied to smooth muscle cells at certain beats per minute. These conditions not only provided mechanical support, but also mimicked the pulsation of blood in vessels. After maturing in the bioreactor for a week, smooth muscle cells appeared as elongated cells with circumferential or longitudinal orientations. Finally, fibroblasts were wrapped around smooth muscle cells to form an outer membrane, and endothelial cells were planted on the lumen surface. The whole process took nearly 3 months. Compared with the bursting pressure of the saphenous vein, the bursting pressure of this vascular graft was more than 2,500 mmHg, with better mechanical properties. The vascular graft was implanted in 10 patients with end-stage renal disease as a hemodialysis pathway. Three months post-implantation, the patency rate was 60% (McAllister et al., 2009; L'Heureux et al., 2007).

**FIGURE 4:**
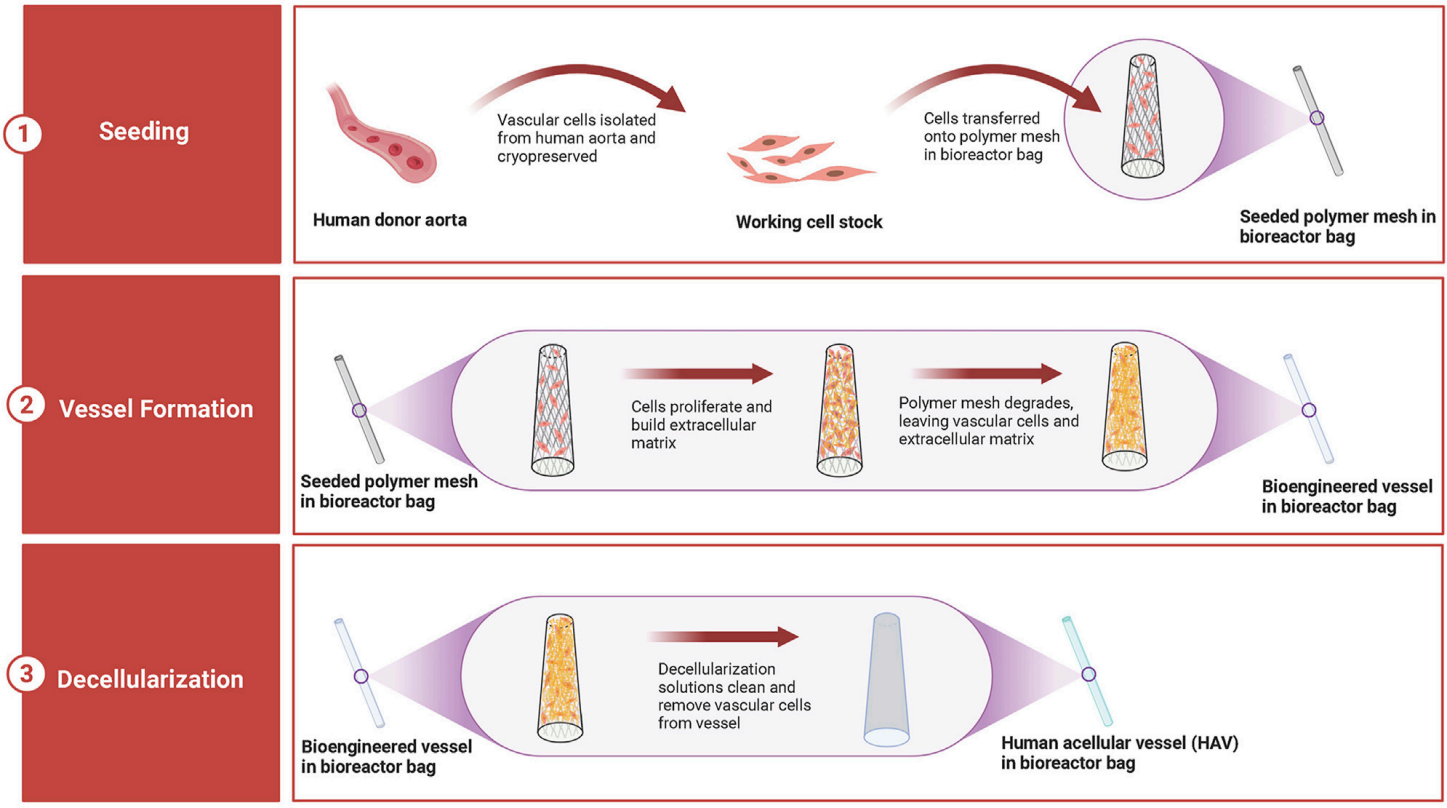
Production of human acellular vessels (HAVs). HAVs are prepared in the bioreactor by seeding human donor vascular cells onto biocompatible and biodegradable mesh scaffolds. In a few weeks, cells grow and produce new tissues, which form tubular vascular structures, while the mesh scaffolds degrade. Then, the generated vessels are decellularized to remove the immunogenicity and components that may induce an immune response. After decellularization, the extracellular matrix structure of HAVs is complete, and the biomechanical properties of blood vessels are also retained. HAVs stored in the bioreactor can be easily transported and used immediately when needed.

In 1999, (Niklason et al., 1999), were the first to describe the latest preparation process of tissue-engineered arteries. PGA scaffold, which degraded rapidly, was wrapped around the silicone tube and placed in the bioreactor. In the bioreactor, smooth muscle cells were planted on the PGA scaffold, and mechanical stimulation of vascular pulsation and stretching was simulated. After 8 weeks of culturing in the bioreactor, the matrix secreted by smooth muscle cells was deposited on the scaffold, and PGA was degraded. The generated tissues were decellularized to generate decellularized vascular matrix. This technology is used to produce human acellular vessels (HAVs) (Figure 4). It is also being tested for hemodialysis access and vascular reconstruction following trauma.

Bioreactors for the production of TEVGs can be designed for different functions and types in accordance with research purposes (Supplementary Figure S1). The main body of the bioreactor is a glass reservoir fitted with a silicone stopper. The vessels are cannulated with glass pipettes, secured with silk sutures, and placed into the bioreactor. Silicone tubing is attached to the glass pipettes, reservoir inlet, and outlet to complete the perfusion loop. The flow rate, intramural pressure, and wall shear stress can be tuned effectively by adjusting various conditions, for instance, pump speed, tubing resistance, media viscosity, and so on.

Since these groundbreaking studies, significant breakthroughs have been made in the research on vascular grafts using tissue engineering techniques. Some of these have shown promising results in animal models; nonetheless, supporting clinical research data is still required.

## 5 Decellularization technologies in decellularized vascular matrix

When donor cells enter the receptor who receives the treatment of vascular grafts, they can induce inflammation and immune-mediated rejection as foreign antigens. Decellularization technology involves a variety of methods to remove cells and nucleic acids, which usually cause immune rejection, and retain the extracellular matrix. Extracellular matrix obtained by decellularization techniques is used to construct tissue engineering vascular scaffold. It can facilitate the acceptance of the vascular graft by the host and avoid rejection after implantation.

Ideally, after decellularization treatment, the cellular components of the vessel should be completely removed, and the extracellular matrix can be retained to the maximum extent. The vessel should still have good mechanical properties after decellularization. Moreover, since decellularization reagents often contain toxic substances, residual toxic substances in the protocol need to be removed post-decellularization to ensure the biocompatibility of the vascular matrix.

Currently, studies have reported the use of physical, chemical, and biological methods, or their combination, all of which have achieved more desirable decellularization results. Among these, physical methods include freeze-thaw, high hydrostatic pressure, and mechanical agitation; chemical methods include ionic, non-ionic, and amphoteric detergents; and biological methods include using trypsin to digest the cell structure (Gilbert et al., 2006; Crapo et al., 2011; Gilpin and Yang, 2017). We describe here the evaluation criteria for the effectiveness of the techniques for decellularization and summarize the respective advantages and shortcomings of different decellularization techniques.

### 5.1 Evaluation of the decellularization effect

Different decellularization techniques often provide different decellularization results. Post-decellularization, a small amount of residual cellular structure remains, making it difficult to obtain decellularized vessels comprising entirely of extracellular matrix. Hence, a set of reasonable and sound evaluation criteria are needed to judge the effectiveness of decellularization protocols. DNA quantification and qualitative evaluation of cell removal are common methods (Naso and Gandaglia, 2022). Particularly, Gilbert et al. (2006), Crapo et al. (2011), and Keane and Badylak (2014) have conducted numerous trials in different animals and tissues and have proposed the following evaluation criteria for the effectiveness of decellularization: <50 ng/double-stranded (ds) DNA/mg extracellular matrix dry weight; <200 bp DNA fragmented length; Lack of visible nuclear materials after staining either with 40,6-diamidino-2-Phenylindole or hematoxylin and eosin. However, the effect of cell residues on host response is not only caused by DNA, but may be the result of the interaction of multiple structures. Therefore, we summarize a set of common but comprehensive evaluation criteria and test methods for the decellularization effect (Table 2) (Naso and Gandaglia, 2022).

**TABLE 2:** Common test methods for evaluation of the decellularization effect.

Evaluation index	Characterization modes	Test methods	Details
DNA	Quantitative	Commercial Kit	For DNA extraction and quantification
Nucleus	Localization	Immunofluorescence assessment and Histological staining	4′,6-diamidino-2-phenylindole (DAPI), Ematoxylin and Eosin
Collagen	Qualitative	Immunofluorescence assessment and Histological staining	Picro Sirius Red, Masson, Mallory and Van Gieson Trichromic staining and anti-collagen I, II, and III antibodies
	Quantitative	Experiments	Sodium dodecyl sulfate-polyacrylamide gel electrophoresis (SDS–PAGE), High-performance liquid chromatography with fluorescence detection (HPLC-FLD)
Elastin	Qualitative	Immunofluorescence assessment and Histological staining	Verhoeff and anti-elastin antibodies
	Quantitative	Experiments	NaOH extraction, Desmosine-base assay
Structural cell proteins	Qualitative	Immunofluorescence assessment	α-Smooth Muscle Actin (αSMA), β-Actin, Vimentin
Antigens	Qualitative	Immunohistochemistry assessment	Anti-Neu5Gc, anti α-Gal, and anti-SDa antibodies and Major Histocompatibility Complex (MHC)
	Quantitative	ELISA	

### 5.2 Physical decellularization techniques

The advantage of physical decellularization is the relative simplicity of the procedure. Nevertheless, if the goal is to minimize the destruction of the extracellular matrix, physical decellularization cannot be used alone and needs to be used in combination with other decellularization protocols to achieve good results (Table 3).

**TABLE 3:** Physical treatments used for decellularization.

Category	Definition	Advantages	Shortcomings	References
Freeze-thaw	An ice crystal structure is formed to increase cytoplasm concentration and cause cell dissolution	-Less effective on the structural and mechanical properties	-Still contains cell components	Teo et al. (2011); Lu et al. (2012); Burk et al. (2014); Cheng et al. (2019); Fernandez-Perez and Ahearne (2019)
		-Significant effect of chemical methods after freeze-thaw	-Requires further decellularization by chemical methods or enzymes	
High hydrostatic pressure	The cell membranes are damaged by deformation under pressure higher than 600 MPa	-No chemical reagent added	-Damages the collagen and elastin to affect mechanical properties	Diehl et al. (2005); Morimoto et al. (2015); Morimoto et al. (2017); Kurokawa et al. (2021); Matsuura et al. (2021)
		-Avoids damage from the toxic effects of solvents		
Supercritical fluids	Cell residues can be removed when supercritical carbon dioxide passes through tissues at a controlled rate similar to critical point drying	-Less impact on the mechanical properties	-Poor solubility for macromolecules and polar substances	Kim et al. (2013); Huang et al. (2021); Kim et al. (2021); Reis et al. (2022)
		-Much simpler procedure	-Easily introduces new impurities after adding entrainment agents to improve solubility	
Mechanical Agitation	Uses a magnetic stirring tray or shaker throughout the decellularization process	-Facilitates the full contact and penetration of chemicals into the tissue	-Poor effect when used singly	Yang et al. (2010); Syedain et al. (2011); Sarig et al. (2012); Boriani et al. (2017); Duisit et al. (2018)
		-Significantly improves the efficiency		

### 5.3 Chemical decellularization techniques

The chemical decellularization techniques include a variety of reagents and is the main choice among current decellularization approaches (Table 4). Nonetheless, different reagents bring about different decellularization effects. For example, mild reagents are less destructive to the extracellular matrix, while the effects of powerful reagents are the opposite. Accordingly, the reagents should be carefully chosen after combining the structure and properties of the material to be decellularized.

**TABLE 4:** List of chemical treatments used for decellularization.

Category	Definition	Advantages	Shortcomings	References
Hypotonic or hypertonic buffer	Changes cellular osmolarity	-Hypotonic buffer can lead to cell swelling and damage the minimal structure	-Needs to be combined with other chemical technologies in most cases	Wainwright et al. (2010); Yang et al. (2010); Kim et al. (2016); Vafaee et al. (2018)
		-Hypertonic buffer can dissociate DNA from proteins		
Acid or base	Application of acid or base to lyse the cytoplasmic components	-Removes nucleic acid and other substances	-Denatures proteins to affect their structure and function	Wainwright et al. (2010); Syedain et al. (2014); Mimler et al. (2019)
			-NaOH can lead to fracture and degradation of collagen fibers within the vascular matrix	
Non-ionic detergent	A mild detergent that disrupts interconnections between DNA, proteins, and lipids, thereby disrupting cell structure	-Maintains the ultrastructure	-More toxic. For example, TritonX-100 easily leads to cell death and needs to be washed thoroughly after decellularization	Wainwright et al. (2010); Yang et al. (2010); Lu et al. (2012); Xu et al. (2014); Baert et al. (2015); Kim et al. (2016); Daugs et al. (2017); Huh et al. (2018); Simsa et al. (2018); Cheng et al. (2019); Fernandez-Perez and Ahearne (2019); Laker et al. (2020); Marin-Tapia et al. (2021)
		-Retains the biological activity effectively		
Ionic detergent	A powerful detergent that destroys cell membranes and completely denatures proteins	-Effective in dissolving membranes, lipids, and DNA.	-Causes disruption of collagen integrity and loss of glycosaminoglycans	Wainwright et al. (2010); Pan et al. (2014); Xu et al. (2014); Baert et al. (2015); Daugs et al. (2017); Huh et al. (2018); Simsa et al. (2018); Vafaee et al. (2018); Cheng et al. (2019); Fernandez-Perez and Ahearne (2019); Laker et al. (2020); Marin-Tapia et al. (2021)
Amphoteric ionic detergent	A relatively mild decellularization reagent and the effect between non-ionic detergent and ionic detergent	-SB-10 and SB-16 exhibit better preservation of the extracellular matrix and better cell removal compared to non-ionic detergents	-Tends to cause the denaturation of the protein structure	Kim et al. (2016); Daugs et al. (2017); Simsa et al. (2018); Bae et al. (2021); Marin-Tapia et al. (2021)
		-CHAPS retains more collagen, glycosaminoglycans, and elastin compared to ionic detergents		

### 5.4 Biological decellularization techniques

Biological decellularization techniques are currently less diverse and seem to be less developed, and are less effective when used alone (Table 5). Thus, decellularization through a biological approach is usually performed in combination with chemical decellularization reagents to achieve satisfactory results similar to those obtained by physical decellularization techniques.

**TABLE 5:** List of enzymes used for decellularization.

Category	Definition	Advantages	Shortcomings	References
Trypsin	Selectively cleaves the cell adhesion protein on the carboxyl side of arginine or lysine to detach cells from the tissue surface and destroys the extracellular matrix around the collagen fiber to generate small channels and promote penetration	-Too strong decellularization effect and is required in low concentrations	-Easier to destroy the structure in large quantities	Xu et al. (2014); Giraldo-Gomez et al. (2016); Huh et al. (2018); Lin et al. (2018); Rahman et al. (2018)
Nuclease	Endonucleases, including deoxyribonuclease and ribonuclease, hydrolyze the deoxyribonucleic acid chain and ribonucleic acid chain, respectively	-Helps remove residual nucleic acid added to the detergent	-May destroy the mechanical stability	Mancuso et al. (2014); Mangold et al. (2015); Simsa et al. (2018); Vafaee et al. (2018); Bae et al. (2021)
			-Reduces the glycosaminoglycans	

## 6 Modification methods of decellularized vascular matrix

Despite the above-mentioned advantages of the decellularized vascular matrix, such as superior cytocompatibility and non-immunogenicity, it still suffers from post-transplant vascular lumen thrombosis, neoplastic endothelial proliferation, and weak physical and mechanical properties. As a consequence, researchers worldwide have tried various modification methods to improve the decellularized vascular matrix.

There are various modification methods for decellularized vascular matrix, which can be broadly classified into physical, chemical, and biological methods of modification. Among them, biological modification methods for the construction of artificial blood vessels are currently receiving increasing attention owing to their excellent biocompatibility.

### 6.1 Physical modification methods

Liu and Zhang (2016) observed that the polyelectrolyte multilayers constructed with heparin and SDF-1α bind firmly to the surface of decellularized blood vessels in rats. Besides good hemocompatibility and anticoagulation, these polyelectrolyte multilayers could significantly enhance the migration ability of rat bone marrow MSCs and promote the recellularization of vascular grafts.

Huang et al. (2008) subjected decellularized vascular matrix to vacuum thermal cross-linking treatment; the process improved the mechanical strength and biological stability of the decellularized vascular matrix attributed to inter- and intramolecular dehydration of collagen and the formation of urethane bonds, concurrently maintaining a certain degree of hydrophilicity and porosity.

Xia et al. (2009) treated decellularized rabbit abdominal aorta with plasma and observed a significant reduction in contact angle, inhibition of platelet adhesion, and increased hydrophilicity of the decellularized vascular matrix.

### 6.2 Chemical modification methods

The most commonly used chemical cross-linking agents are glutaraldehyde (GA) and 1-ethyl-3-(3-dimethylaminopropyl) carbodiimide hydrochloride (EDC)/n-hydroxysuccinimide (NHS).

GA is the most commonly used cross-linking modification reagent in collagen-based tissue engineering materials due to its low cost and high efficiency. Compared to uncross-linking decellularized vascular matrices, vascular grafts with GA cross-linking show greater improvements in mechanical properties and immunogenicity. However, with the increase in the cross-linking time, the cytotoxic effects of chemical cross-linking agents become apparent and are more likely to cause tissue calcification (Braile et al., 2011; Sinha et al., 2012).

EDC cross-linking decellularized vascular matrices have better biocompatibility and higher cell differentiation potential compared to those obtained by GA treatment. These vascular matrices obtained after EDC treatment are catalytic; thus, EDC/NHS is also gradually and widely used for the chemical cross-linking of collagen biomaterials. Among them, NHS is used to stabilize the crosslinking effect of carbodiimide (Olde Damink et al., 1996).

### 6.3 Bio-modification methods

Heparin is used to prevent thrombosis because it inhibits the thrombin coagulation cascade reaction. (Conklin et al., 2002) could inhibit thrombus formation by bridging heparin on decellularized treated porcine carotid arteries.

Zhang et al. (2010) used the sheep forelimb artery for acellular treatment, applied the light crosslinking method to fix the CD34 antibody on the inner surface of the acellular vessel, and then implanted it into the rabbit femoral artery. This experiment showed that the CD34 antibody-modified decellularized vascular scaffold was superior to the unmodified decellularized vascular scaffold in terms of early anticoagulation and patency rate.

Fu et al. (2021) introduced Silk Fibroin into decellularized vascular scaffolds to improve the physical properties of the scaffolds and also assessed the performance of the scaffolds. They found that Silk Fibroin effectively improved the overall mechanical properties of the stent and reduced the stent degradation rate.

With advances in biology, chemistry, and materials sciences, the improvement in performance and long-term patency by applying various modifications in the decellularized vascular matrix has positive implications, while the effects have yet to be validated in clinical trials.

## 7 Commercialization process of decellularized vascular matrix

The two most common artificial vascular grafts commercially available today are prepared from common plastics, namely ePTFE (called Gore-Tex) and polyethylene terephthalate (PET; called polyester). Both vascular grafts are robust and can be easily stored for ready access. Furthermore, they can be mass-produced to the desired diameter and length specifications.

**FIGURE 5:**
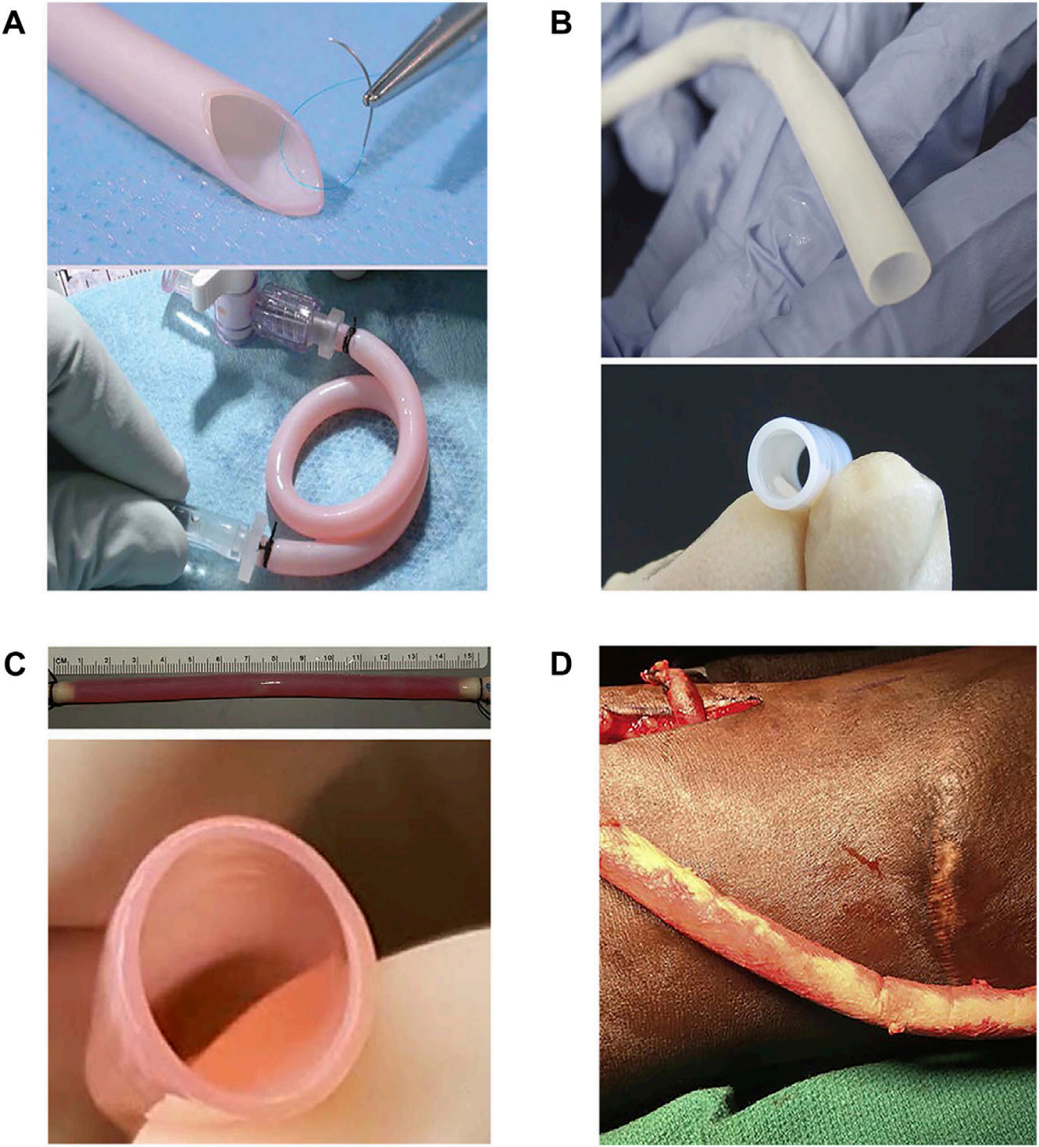
Representative products of four companies. **(A)** Cytograft Tissue Engineering Inc.—The Lifeline™ graft: a completely biological, living, and autologous human blood vessel. The Lifeline™ graft displays remarkably high burst pressure, even though it contains no scaffolding materials, and also shows good suture retention abilities as well as remarkable flexibility (L’Heureux and McAllister, 2008). **(B)** Humacyte, Inc., —Human acellular vessels (HAVs): gross photos of engineered human acellular vessels, 6 mm in diameter (Niklason and Lawson, 2020). **(C)** Vascudyne, Inc. —Arteriovenous graft (AVG): side view and end-on view image of a 6-mm-diameter decellularized tissue-engineered vascular graft (Syedain et al., 2017). **(D)** LeMaitre Vascular, Inc., —Artegraft^®^: a bovine carotid artery (BCA) graft before tunneling (Pineda et al., 2017).

Nonetheless, these synthetic materials are more rigid than natural blood vessels. Except for rough material surfaces, they are highly hydrophobic and not able to replicate the key structures and components of natural vessel walls. These characteristics result in poor interaction with various cells in the blood and predispose them to activate the blood coagulation cascade (Dekker et al., 1991; Filipe et al., 2018; Bao et al., 2020). Compared to synthetic materials, decellularized vascular matrices have a broader commercial prospect as vascular grafts, being highly cytocompatible and non-immunogenic. Therefore, many companies around the world are already trying to prepare decellularized vascular matrices as vascular grafts and are working to advance their commercialization. Four well-known corporations and their representative productions are briefly introduced here (Figure 5; Table 6).

**TABLE 6:** Properties comparison of the representative products of four companies.

Company	Graft	Burst pressure (mmHg)	Suture retention strength (gf)	Ultimate tensile strength (MPa)	Compliance (%)	Primary patency
Cytograft Tissue Engineering Inc.	Lifeline™	3,486 ± 500	162 ± 15	Not mentioned	1.5 ± 0.3	66.7% for 13 months
Humacyte, Inc.	Human acellular vessels (HAVs)	2,914 ± 928	440 ± 85	Not mentioned	Not mentioned	89% for 1 year
Vascudyne, Inc.	Arteriovenous graft (AVG)	3,164 ± 342	199 ± 56	3.8 ± 0.7	Not mentioned	60% for 6 months
LeMaitre Vascular, Inc.	Artegraft^®^	2,422 ± 354	Not mentioned	Not mentioned	3.3 ± 0.9	73.3% for 1 year

### 7.1 Cytograft tissue engineering Inc

Cytograft Tissue Engineering Inc., was one of the early companies to develop artificial vessels and The Lifeline™ graft as a typical product. Early research into the use of cell sheet engineering to reconstruct artificial vessels gave rise to the idea of developing “human textiles” (L'Heureux et al., 2006). Small biopsy skin samples of skin and superficial veins were obtained, and endothelial cells and fibroblasts were extracted. Fibroblast sheets were produced in as little as 6 weeks. The obtained TEVGs consisted of three components: a living adventitia, a decellularized internal membrane and an endothelium. This procedure cost upwards of $15,000 and required 6–9 months to produce patient-specific vascular grafts. However, it is unlikely that this vascular graft can be applied clinically owing to high production costs and long waiting times. Despite all this, its method of preparation provided a new idea for research on artificial vascular grafts.

### 7.2 Humacyte, Inc

**FIGURE 6:**
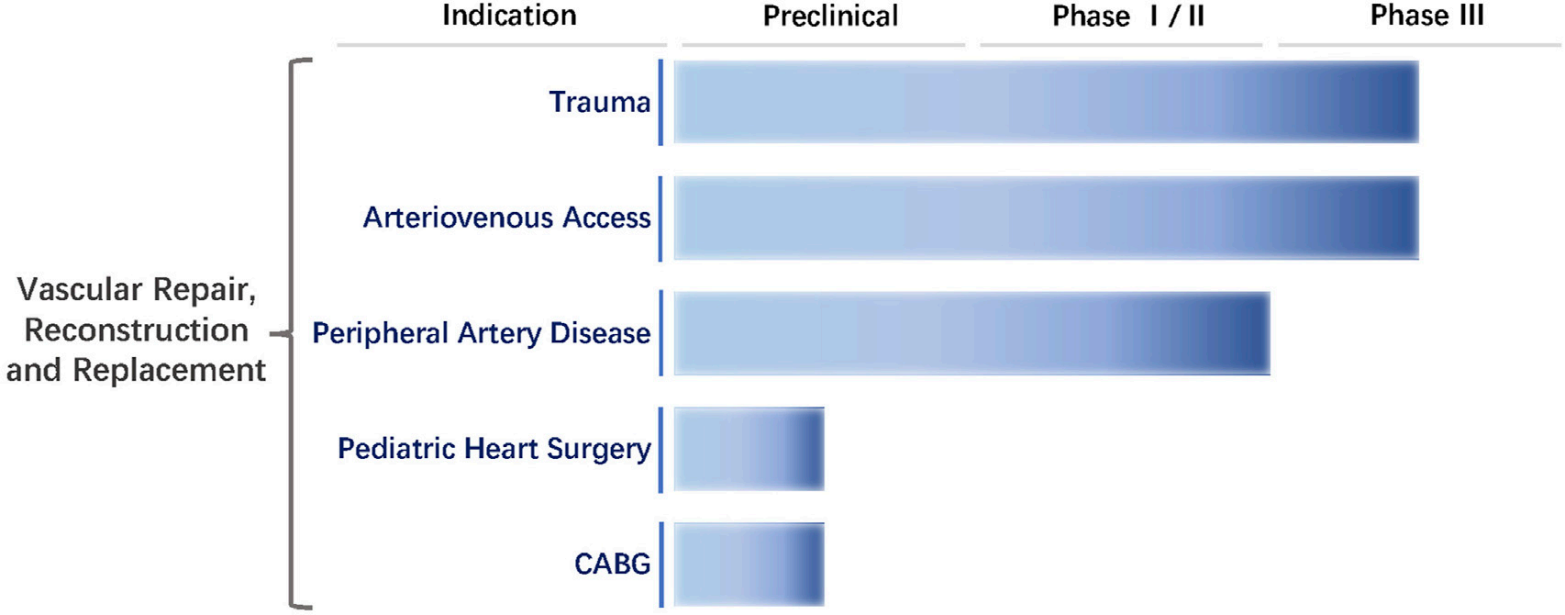
Clinical trials of Humacyte, Inc. The clinical trials conducted by the company for the treatment of trauma and arteriovenous access are in Phase Ⅲ. Peripheral vascular bypass grafting has completed Phase Ⅱ. Pediatric heart surgery and coronary artery bypass grafting (CABG) are in the preclinical trials.

Humacyte, Inc., was founded in 2004 by Laura E. Niklason of Yale University. The firm aims to develop innovative technologies for regenerative vessels and focuses on a new type of vascular graft known as HAVs (Dahl et al., 2011). HAVs are completely decellularized and mechanically robust with circumferentially aligned extracellular matrix without any chemical cross-linking or synthetic components (Niklason and Lawson, 2020). They have been favorably repopulated by host cells in multiple advanced-stage clinical trials in vascular trauma repair, arteriovenous access for hemodialysis, and peripheral arterial disease. In eight clinical trials to date, HAVs have been implanted in more than 500 patients. The firm is currently conducting three Phase Ⅲ clinical trials, one Phase Ⅱ clinical trial, and three Phase Ⅰ clinical trials worldwide (Kirkton et al., 2019; Lauria et al., 2022) (Figure 6).

### 7.3 Vascudyne, Inc

Vascudyne, Inc., was founded by Jeff Franco and Kem Schankereli. It licensed its proprietary TRUE™ Tissue technology, developed in 2017 by Professor Robert Tranquillo and his colleagues from the University of Minnesota (Supplementary Figure S2). The current products are TRUE™ Tissue, including TRUE™ Graft, TRUE™ Valve, and TRUE™ Patch (Syedain et al., 2021). The biological and mechanical properties of TRUE™ Graft are very similar to those of native tissue; preclinical studies have shown that TRUE™ Graft regenerate and grow with the patient. In July 202, Vascudyne, Inc., announced the first successful in-human use of its TRUE™ Vascular Graft in patients with the end-stage renal disease requiring hemodialysis access.

### 7.4 LeMaitre vascular, Inc

Artegraft^®^ approved by FDA in 1970, is a bovine carotid vascular graft. Its decellularized matrix is treated for use as hemodialysis access and lower extremity bypass. The biological fibrous matrix of this graft has been processed to enhance long-term patency and provides a tightly woven, cross-linking conduit that is flexible and compliant. During the past 50 years, Artegraft^®^ has been implanted in more than 5,00,000 patients.

In October 2020, Hancock Jaffe Laboratories, Inc., (HJLI) announced the first use of grafts in CABG. The first-in-human CoreoGraft study of HJLI was completed, and the patient was discharged from the hospital. In May 2022, Medical 21, Inc., announced that they have been developing an artificial graft called MAVERICS, a small-diameter flexible tube encased in a nickel-titanium alloy stent that eliminates the need to harvest blood vessels from the patient’s legs, arms, and chest. The goal is to improve the quality of life of patients by reducing pain, shortening surgery and recovery time, and reducing the risk of infection and complications.

As already stated, these four representative corporations have been working towards the commercialization process of the decellularized vascular matrix as vascular grafts. Vascular grafts are class III implantable biological medical devices in the International Organization for Standardization (ISO), the US Food and Drug Administration (FDA) and EU classification standards. ISO is an independent non-governmental organization that has created thousands of international standards for many industries, including medical equipment. ISO standards are voluntary, consensus-based documents that provide guidance on specific aspects of technology and manufacturing. For medical device manufacturers, ISO standards are not only important for building high-quality medical devices, but also for maintaining compliance with regulatory requirements. Most of the ISO standards have been recognized by regulatory agencies such as FDA, or have been harmonized with regulations in other regions of the world (such as the EU). Even if ISO standards do not have legal force, they are also basic guidelines for medical devices and *in vitro* diagnostic equipment companies. We list the ISO Standard aspect to which commercial vascular grafts are subject before they can be put on the market (Supplementary Table S1). In addition, commercial decellularized vascular grafts need to be accord with an international standard publishes in 2021 (ASTM International, 2021). The commercialization process of vascular grafts is tortuous and arduous; in terms of clinical application, it still has a long way to go. For example, the research on product development is not mature enough, and the clinical trials have not been completed yet. On the whole, the commercialization process of vascular grafts is still improving and advancing. Through the active efforts of researchers across the world, the clinical needs of vascular grafts are expected to be met in the near future.

## 8 Conclusions and prospects

**FIGURE 7:**
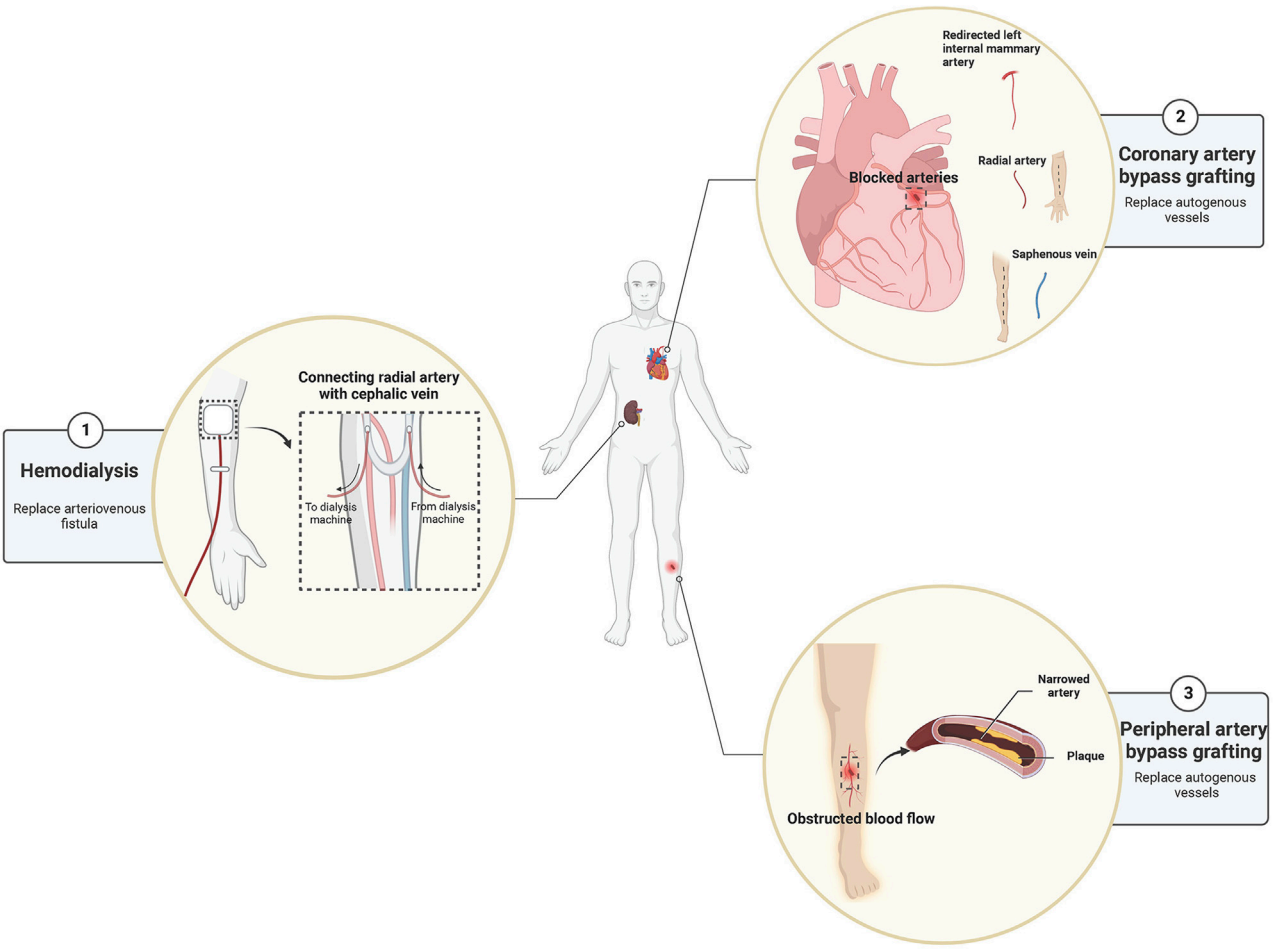
The prospects of the use of the decellularized vascular matrix in small-diameter vascular grafts.

Although the research on the decellularized vascular matrix in tissue engineering has progressed significantly, small-diameter vascular graft still faces the risks of infection, restenosis, and thrombosis after implantation for some time to come. Among them, post-implantation restenosis due to insufficient endothelialization is the main reason why small-diameter vascular graft such as decellularized vascular matrix is difficult to use clinically. Despite these drawbacks, the decellularized vascular matrix is still considered an ideal choice for vascular grafts and has promising application prospects. For example, in CABG surgery, decellularized vascular grafts benefit a vast number of cardiovascular patients. Besides its application in CVD, the decellularized vascular matrix is also widely used to treat other diseases, for example, arteriovenous bypass grafting in patients with the end-stage renal failure before hemodialysis and peripheral vascular bypass grafting with peripheral vascular damage due to trauma (Figure 7).

Promoting rapid endothelialization of the lumen of vascular grafts is the key to improving the patency rate. The ultimate goal of promoting endothelialization is to inhibit the formation of thrombus and the proliferation of neointima. By using various technologies, such as surface modification and cross-linking treatment, or multiple materials to optimize the material ratio, vascular grafts are endowed with better mechanical, biological and immunological properties.

However, the relevant technologies across the globe are not mature enough. For example, in addition to the exact effect of the current decellularization technologies, the long-term efficacy and safety of foreign substances, such as modified materials, in the human body have not been widely confirmed. This requires clinical trials and follow-ups for a long time as an effective and reliable basis for judgment. Consequently, the application of decellularized vascular matrix in small-diameter vascular grafts has a long way to go before it can be realized. To solve this series of problems, there is a need for cooperation between materials science, tissue engineering, biology, and other disciplines to jointly promote the development of tissue engineering. In this way, small-diameter artificial blood vessels such as decellularized vascular matrix can be used better in vascular transplantation.
