# Supplementary figures and images for: Application of decellularized vascular matrix in small-diameter vascular grafts
Source: Front Bioeng Biotechnol. 2023 Jan 6;10:1081233. doi: 10.3389/fbioe.2022.1081233 (PMC9852870; doi:10.3389/fbioe.2022.1081233)

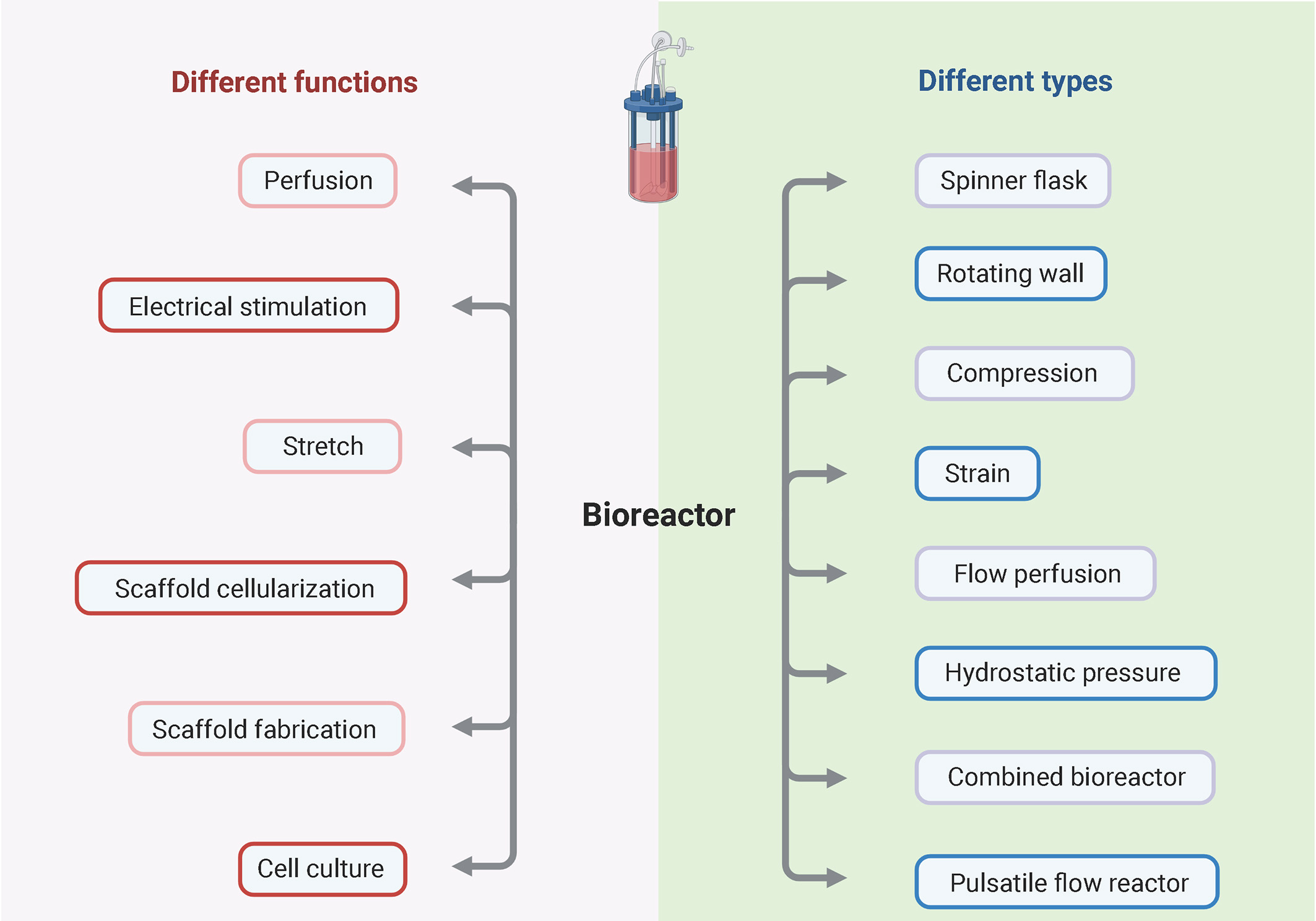

Supplement: Supplementary file 1 [file Image1.JPEG]

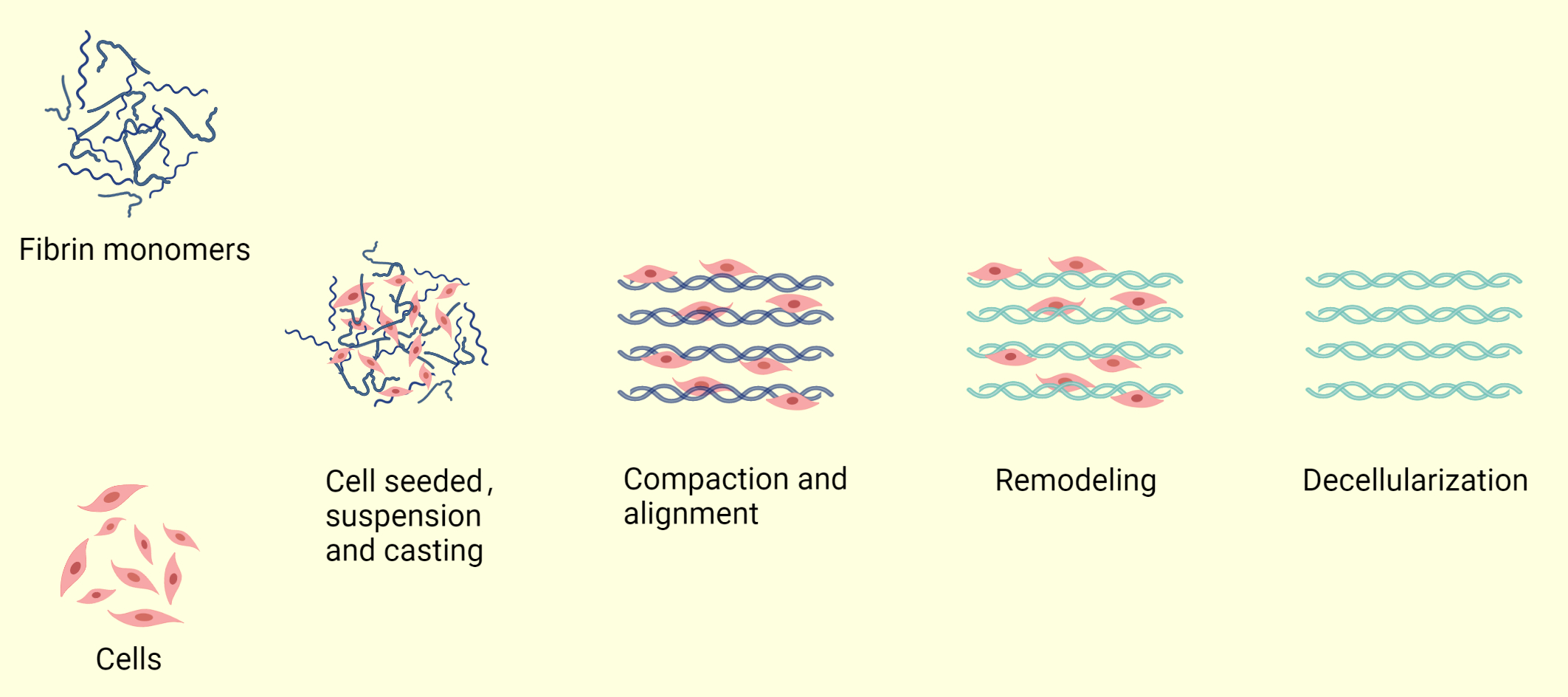

Supplement: Supplementary file 2 [file Image2.JPEG]
