# Supplementary material for: Application of decellularized vascular matrix in small-diameter vascular grafts
Source: Front Bioeng Biotechnol. 2023 Jan 6;10:1081233. doi: 10.3389/fbioe.2022.1081233 (PMC9852870; doi:10.3389/fbioe.2022.1081233)
Supplement: Supplementary file 3 [file DataSheet1.docx]

Supplementary Material

Application of decellularized vascular matrix in small-diameter vascular grafts

Yuanming Li^1^, Ying Zhou^1^, Weihua Qiao^1^, Jiawei Shi^1^, Xuefeng Qiu ^1*^, Nianguo Dong ^1*^

^1^Department of Cardiovascular Surgery, Union Hospital, Tongji Medical College, Huazhong University of Science and Technology, Wuhan, China

*** Correspondence:**Xuefeng Qiu
[qxf027@163.com](mailto:qxf027@163.com)

Nianguo Dong
[dongnianguo@hotmail.com](mailto:dongnianguo@hotmail.com)

# Supplementary Figures and Tables

## Supplementary Figures

### Supplementary Figure S1. Different functions and types of the bioreactor for the production of tissue-engineered vascular grafts.


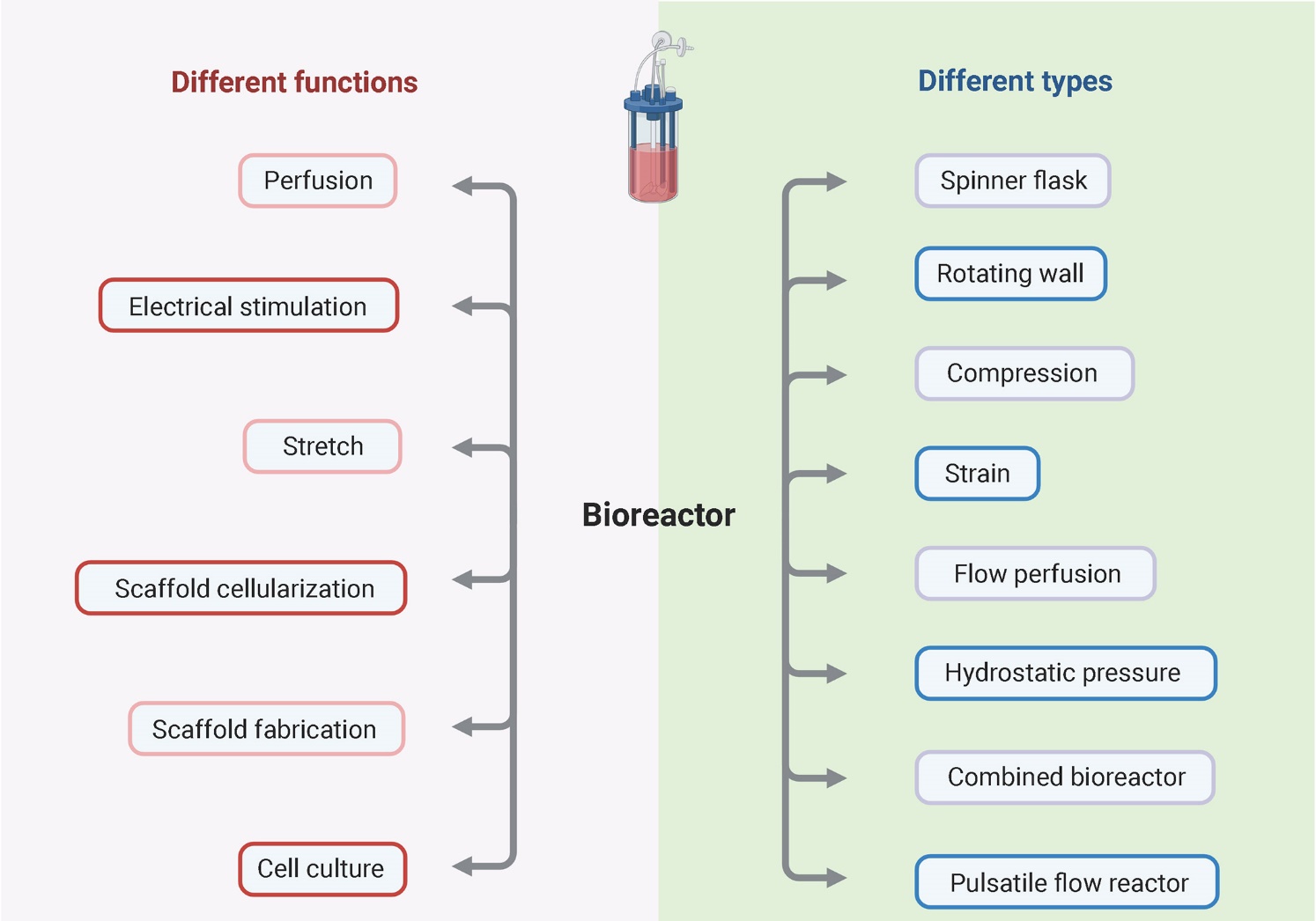


### Supplementary Figure S2. TRUE™ Tissue Technology (Vascudyne, Inc).


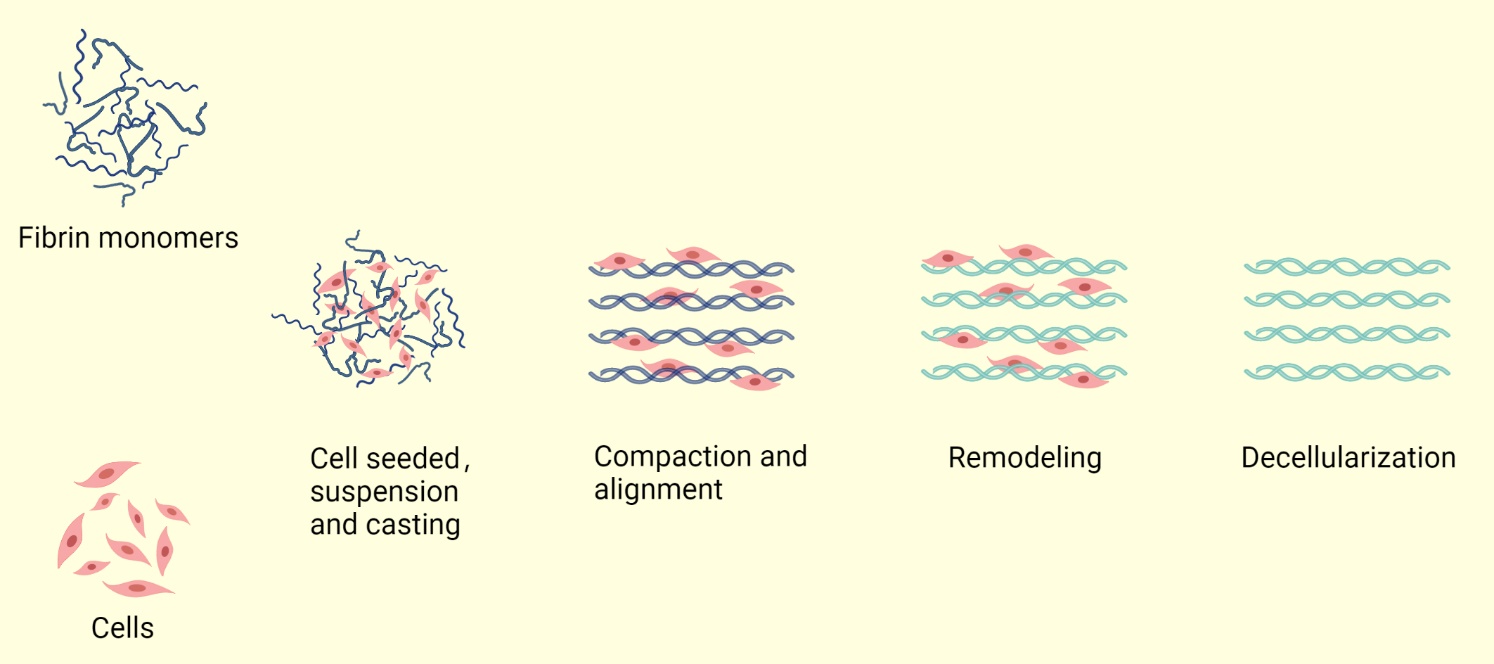


## Supplementary Tables

### Supplementary Table S1. ISO Standards for commercial decellularized vascular grafts.

| No. | Subtitle | Aspect |
| --- | --- | --- |
| ISO 10993 | Biological evaluation of medical devices | Biological evaluation |
| ISO/TS 37137 | Biological evaluation of absorbable medical devices |  |
| ISO/TS 21726 | Biological evaluation of medical devices — Application of the threshold of toxicological concern (TTC) for assessing biocompatibility of medical device constituents |  |
| ISO/TR 37137 | Cardiovascular biological evaluation of medical devices — Guidance for absorbable implants |  |
| ISO 25539 | Cardiovascular implants — Endovascular devices | Cardiovascular implants |
| ISO/TS 17137 | Cardiovascular implants and extracorporeal systems — Cardiovascular absorbable implants | Cardiovascular implants and extracorporeal systems |
| ISO 7198 | Cardiovascular implants and extracorporeal systems — Vascular prostheses |  |
| ISO 14155 | Clinical investigation of medical devices for human subjects — Good clinical practice | Clinical investigation |
| ISO/TS 21560 | General requirements of tissue-engineered medical products | General requirements of tissue-engineered medical products |
| ISO/TR 14283 | Implants for surgery — Essential principles of safety and performance | Implants for surgery |
| ISO 16054 | Implants for surgery — Minimum data sets for surgical implants |  |
| ISO 16428 | Implants for surgery — Test solutions and environmental conditions for static and dynamic corrosion tests on implantable materials and medical devices |  |
| ISO 14971 | Medical devices — Application of risk management to medical devices | Application of risk management |
| ISO 20417 | Medical devices — Information to be supplied by the manufacturer | Information to be supplied by the manufacturer |
| ISO/TR 20416 | Medical devices — Post-market surveillance for manufacturers | Post-market surveillance |
| ISO 13485 | Medical devices — Quality management systems — Requirements for regulatory purposes | Quality management systems |
| ISO 16142 | Medical devices — Recognized essential principles of safety and performance of medical devices | Recognized essential principles |
| ISO 15223 | Medical devices — Symbols to be used with medical device labels, labelling, and information to be supplied | Symbols |
| ISO 22442 | Medical devices utilizing animal tissues and their derivatives | Utilizing animal tissues and their derivatives |
| ISO 14630 | Non-active surgical implants — General requirements | Non-active surgical implants |
| ISO 17327 | Non-active surgical implants — Implant coating |  |
| ISO 11607 | Packaging for terminally sterilized medical devices | Packaging |
| ISO/TS 16775 | Packaging for terminally sterilized medical devices |  |
| ISO 17664 | Processing of health care products — Information to be provided by the medical device manufacturer for the processing of medical devices | Processing |
| ISO 11140 | Sterilization of health care products — Chemical indicators | Sterilization |
| ISO 20857 | Sterilization of health care products — Dry heat |  |
| ISO 14937 | Sterilization of health care products — General requirements for characterization of a sterilizing agent and the development, validation and routine control of a sterilization process for medical devices |  |
| ISO 14160 | Sterilization of health care products — Liquid chemical sterilizing agents for single-use medical devices utilizing animal tissues and their derivatives |  |
| ISO 25424 | Sterilization of health care products — Low temperature steam and formaldehyde |  |
| ISO 22441 | Sterilization of health care products — Low temperature vaporized hydrogen peroxide |  |
| ISO 11737 | Sterilization of health care products — Microbiological methods |  |
| ISO 17665 | Sterilization of health care products — Moist heat |  |
| ISO 11137 | Sterilization of health care products — Radiation |  |
| ISO 11135 | Sterilization of health-care products — Ethylene oxide |  |
| ISO/TS 21387 | Sterilization of medical devices — Guidance on the requirements for the validation and routine processing of ethylene oxide sterilization processes using parametric release |  |
